# Assessment of the cyst wall and surface microbiota in dormant embryos of the Antarctic calanoid copepod, *Boeckella poppei*

**DOI:** 10.1111/1758-2229.70035

**Published:** 2024-11-27

**Authors:** Hunter B. Arrington, Sung Gu Lee, Jun Hyuck Lee, Joseph A. Covi

**Affiliations:** ^1^ Department of Biology and Marine Biology The University of North Carolina at Wilmington Wilmington North Carolina USA; ^2^ Division of Polar Life Science Korea Polar Research Institute (KOPRI) Incheon Korea; ^3^ Department of Polar Sciences University of Science and Technology Incheon Korea

## Abstract

Embryos of zooplankton from inland waters and estuaries can remain viable for years in an extreme state of metabolic suppression. How these embryos resist microbial attack with limited metabolic capacity for immune defence or repair is unknown. As a first step in evaluating resistance to microbial attack in dormant zooplankton, surface colonization of the Antarctic freshwater copepod, *Boeckella poppei*, was evaluated. Scanning electron micrographs demonstrate the outer two layers of a five‐layered cyst wall in *B. poppei* fragment and create a complex environment for microbial colonization. By contrast, the third layer remains undamaged during years of embryo storage in native sediment. The absence of damage to the third layer indicates that it is resistant to degradation by microbial enzymes. Scanning electron microscopy and microbiome analysis using the 16S ribosomal subunit gene and internal transcribed spacer (ITS) region demonstrate the presence of a diverse microbial community on the embryo surface. Coverage of the embryos with microbial life varies from a sparse population with individual microbes to complete coverage by a thick biofilm. Extracellular polymeric substance binds debris and provides a structural element for the microbial community. Frequent observation of bacterial fission indicates that the biofilm is viable in stored sediments.

## Graphical abstract

A diverse microbiome colonizes the surface of dormant copepod embryos, which can survive for ~200 years in lake sediments. During dormancy, the outer two layers of a five‐layered cyst wall break apart, while the third layer remains undamaged, despite the presence of microbial species with chitinase genes.

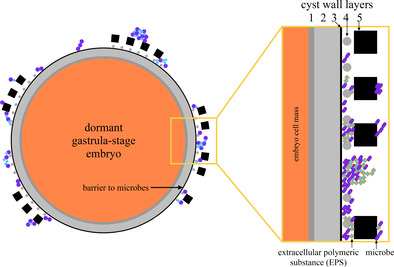

## INTRODUCTION

Dormant embryos of crustacean zooplankton that reside in lake and estuarine sediments must defend themselves against microbial attack with limited or negligible metabolic activity. Zooplankton in benthic environments are capable of extreme dormancy lasting centuries (Frisch et al., 2014; Hairston, 1996; Jiang et al., 2012; Marcus et al., 1994). Metabolic processes must be severely limited for dormant embryos to preserve enough energetic resources to complete development after dormancy. This is pertinent because crustacean exoskeletons are made of one of the most abundant polysaccharides in nature (Khoushab & Yamabhai, 2010) and are susceptible to shell‐disease caused by chitinolytic microbes (Le Bloa et al., 2020; Yurgel et al., 2022). Nearly two centuries of viable dormancy for the chitin‐encrusted copepod, *Boeckella poppei* Mrázek, leads to burial in up to five centimetres of sediment (Jiang et al., 2012). Importantly, hydrolysis of chitin is measurable in up to five centimetres of both oxic and anoxic freshwater sediment (Worner & Pester, 2019). It is unknown how dormant crustacean zooplankton prevent microbial attack while in sediments under a metabolically suppressed state. Colonization by beneficial microbial species could protect the dormant zooplankton from pathogenic microbes with no metabolic cost to the zooplankton. To test hypotheses like this, it is first necessary to determine what microbes reside on the surface of embryos stored in native sediments. That is the aim of this study.

Embryonic dormancy in zooplankton is defined as a cessation of development with or without simultaneous metabolic suppression (Hand et al., 2011). In the most extreme form of dormancy investigated to date, embryos of the brine shrimp, *Artemia franciscana* Kellogg, achieve a nearly ametabolic state with no developmental progression when exposed to anoxia (Hand et al., 2011; Warner & Clegg, 2001). The preservation of energy stores while dormant is critical, because development and hatching after dormancy are energetically expensive. As evidence of this, energy stores are almost completely depleted during development from gastrula to swimming larval stage in the copepod, *B. poppei* (Reed et al., 2021). Thus, metabolism must be severely downregulated during embryonic dormancy in this species of copepod.

Embryonic development and dormancy in *B. poppei* is similar to *A. franciscana* and *Daphnia magna* (Reed et al., 2021). All three species exhibit dormancy under anoxia in the gastrula stage embryo when cells are partially syncytial (Reed et al., 2021). Under anoxia, acidification of the intracellular space and severe depletion of nucleoside triphosphates (NTPs), including ATP, occurs in both the brine shrimp, *A. franciscana* and the copepod, *B. poppei* (Hand, 1998; Reed et al., 2023). The depletion of ATP under anoxia demonstrates that metabolism is downregulated in both species. Extensive studies on brine shrimp embryos also demonstrate that the intracellular acidification acts as a global downregulator of metabolic processes, including protein synthesis (Hand, 1998).

Microbial defences in crustaceans are strictly limited to an innate immune system that is dependent on metabolic activity (Rosa & Barracco, 2010). Microbes degrade crustacean exoskeletons with chitinolytic enzymes (Jimoh et al., 2022; Zhang et al., 2021). Antimicrobial peptides (Matos & Rosa, 2022), c‐type lectins (Du et al., 2022a, 2022b) and chitosan (Bernabé et al., 2020; No et al., 2002) in crustacean hemolymph and cuticle (exoskeleton) provide multiple levels of defence against fungi and prokaryotes. However, it is unknown how dormant zooplankton embryos defend against degradation by microbial enzymes during centuries of dormancy when metabolism is suppressed.

Crustaceans have an external microbiome that is unique and flexible. Epibenthic decapod microbiomes differ both among species within a single site and among geographic sites (Koepper et al., 2023). Microbiomes on crustacean surfaces also vary with the temperature (Kraemer et al., 2020). Limited studies of microbiomes on crustacean embryos also demonstrate selectivity (Eckert & Pernthaler, 2014; Le Bloa et al., 2020). It is plausible that a selective microbiome on dormant zooplankton provides protection against pathogenic microbes.

Antarctic lake systems inhabited by *B. poppei* have simple food webs dominated by microbial life (Laybourn‐Parry & Pearce, 2007, 2016). Microbial community structure within lakes varies substantially with depth, and bacterial biomass is greatest at the sediment/water interface (Laybourn‐Parry & Pearce, 2016). Complex microbial mats often form at the sediment/water interface and consist of viruses, prokaryotes, protists, fungi and microarthropods (Pessi et al., 2018; Pradel et al., 2023; Rochera & Camacho, 2019; Vinocur & Pizarro, 2000). To date, the characterized microbial diversity in maritime Antarctic lakes on King George Island includes viral (Prado et al., 2022), bacterial (Roldan et al., 2022), archaeal (Roldan et al., 2022), periphyton (Camara et al., 2021), fungal (de Souza et al., 2021), protists (Zhang et al., 2022) and soil algae (Rybalka et al., 2023). The exploration of these microbial communities for industrial and medical use is expanding rapidly (Hwengwere et al., 2022). One unexplored source of potentially novel microbes is the surface of dormant crustacean zooplankton. Dormant embryos of copepods, cladocerans and anostracans reside in the benthic environment and are most abundant at the sediment/water interface where microbial diversity (Jiang et al., 2012; Laybourn‐Parry & Pearce, 2016) and chitin hydrolysis (Worner & Pester, 2019) are greatest.

The goal of this study is to examine the diversity of microbial life colonizing the surface of dormant copepod embryos stored in native Antarctic sediments. Understanding microbial diversity on the surface of dormant embryos is the first step in demining how zooplankton embryos passively defend themselves against microbial attack during prolonged dormancy when metabolism is suppressed. The freshwater copepod, *B. poppei*, was selected as a model organism because it is often the only crustacean zooplankton in a simple food web dominated by microbial life in coastal and maritime Antarctic lakes (Laybourn‐Parry & Pearce, 2007, 2016). Consequently, it is possible to definitively identify embryos to species in the sediment.

## EXPERIMENTAL PROCEDURES

### Chemicals

All solutions for the isolation and culturing of embryos were prepared according to Reed et al. (2018). In brief, 0.35‰ artificial freshwater (AFW) and 80% sucrose were prepared with ultra‐pure water according to Reed et al. (2018). All chemicals for microscopy were of electron microscopy grade or higher.

### Sample collection site and storage

Sediment samples were collected from five locations in a single maritime lake (B5) on Barton Peninsula, King George Island, Antarctica, in February 2019, and each sediment grab was considered a single environmental replicate (Table 1). Sediment was collected using a hand‐operated stainless steel grab sampler (Wildco®, Yulee, FL, USA; product number 623‐3110) without disturbing the surrounding sediments. This method retrieves approximately 0.95 L of sediment from the first 5 cm of sediment depth. Individual sediment grab samples were placed in Ziplock bags in the boat during collection and stored in sealed coolers at 0–4°C. Each sediment grab sample was mixed to homogeneity and subdivided into 50 mL aliquots in a field lab at King Sejong Station, King George Island, Antarctica. Each aliquot was stored in a separate Whirl‐pak® bag. All aliquots of a single sediment grab were then placed in a single Ziplock bag, and multiple sediment grab samples were stored together in a single polystyrene box. Care was taken to remove air when sealing bags in Antarctica, and samples were maintained in the dark at 4–8°C during shipping. Samples used in this study were stored at 4°C after shipping and opened only once immediately before use. This storage method limited the reintroduction of oxygen and light until the day of use for experimentation. It also prevented the introduction of non‐native microbes. Samples used for scanning electron microscopy were stored 27–33 months, and samples used for extraction and microbiome sequencing were stored for 38–40 months after collection from the Antarctic lake.

**TABLE 1:** Summary of microbial DNA samples isolated from the surface of dormant embryos of the copepod, *B. poppei*, after storage in native Antarctic lake sediment.

GPS coordinates for unique sediment grab samples taken from a single Antarctic lake	Unique replicates	Embryo abundance (embryos per mL sediment)	Number of embryos used for DNA extraction	DNA recovered (ng)
62°14′23.2″ S 058°44′37.1″ W	M1*	80.40	2412	1590
62°14′23.0″ S 058°44′37.1″ W	M2*	53.50	1605	2460
62°14′23.2″ S 058°44′37.2″ W	M3*	38.83	1359	810
62°14′23.4″ S 058°44′37.4″ W	M4*	28.00	532	1350
62°14′23.2″ S 058°44′37.3″ W	M5^†^	3.93	336	1020

* Only early development (ED) embryos from a single sediment subsample.

^†^ Due to low embryo abundance, multiple sediment subsamples and all embryonic developmental stages were used for extraction of microbial DNA.

### Embryo isolation

Embryos were isolated from sediment using a density‐dependent separation method (Briski et al., 2013; Reed et al., 2018). In brief, sediment containing the embryos was first mixed with 80% sucrose by inversion (5 mL sediment in 45 mL sucrose or 30 mL sediment in 220 mL sucrose). The mixture was then centrifuged at 2000× gravity for 2 min. The pellet was discarded, and the supernatant was then processed using one of two methods. For rinse method #1, the supernatant was filtered by gravity through a 70 μm sterile cell strainer and the retained embryos were rinsed with less than 200–500 mL of sterile 0.35‰ AFW using a sterile 1 mL transfer pipette. The second rinse method (Reed et al., 2018) consisted of filtering the supernatant through a 63 μm sieve that was previously sanitized with 70% isopropanol. Embryos were rinsed on the sieve with 500 mL sterile 0.35‰ AFW. Embryos from both rinse methods were gently transferred into autoclaved collection dishes by passing sterile 0.35‰ AFW through the back of the sieve. Approximately 250 embryos were then selected at random using a stereomicroscope at 20× to 100× magnification for scanning electron microscopy (SEM). *Boeckella poppei* is the only species of crustacean zooplankton present in this study lake, consequently no separation of species was required (Reed et al., 2018, 2021, 2023).

### Scanning electron microscopy

Embryos, which are 100–200 μm in diameter, were segregated into three developmental categories previously characterized by Reed et al. (2018). This includes early development embryos (ED), intermediate development embryos (ID) and pre‐nauplii (PN). All three of these developmental stages occur prior to hatching of the first larval stage. Embryos for each stage were selected at random and fixed for SEM. Three different variations of fixation protocols based on the published methods (Couch et al., 2001; Reed et al., 2021) were used in an attempt to limit the dehydration‐induced collapse of embryos during processing for microscopy.

For the first fixation protocol, specimens were kept in sterile 40 μm cell strainers until critical point drying. Primary fixation was conducted in 0.1 M phosphate buffer (pH 7.4) with 3% glutaraldehyde overnight at 4°C. After primary fixation, specimens were rinsed three times in 0.1 M phosphate buffer (pH 7.4) for 10 min. Secondary fixation was conducted in 0.1 M phosphate buffer (pH 7.4) with 1% osmium tetroxide for 1 h. After secondary fixation, specimens were rinsed three times in 0.1 M phosphate buffer (pH 7.4) for 10 min. Next, specimens were dehydrated by sequential incubation in 20%, 40%, 60%, 80% and 90% ethanol (EtOH) for 10 min at each concentration and 100% EtOH for 15 min. After dehydration, specimens were transferred to porous pots in EtOH and critical point drying was conducted using a Leica EM CPD300 (Wetzlar, Hesse, Germany) with 16 exchanges of liquid carbon dioxide. All specimens were mounted on 3 mm aluminium stubs with a carbon adhesive tab and sputter coated with a Leica EM ACE600 sputter coater (Wetzlar, Hesse, Germany) using 10 nm of gold–palladium, followed by an additional 7 nm of gold–palladium to reduce specimen charging. Some embryos were lost during each step of fixation and critical point drying due to their extremely small size. Six of eleven embryos were retained through this process, and all were analysed by SEM.

The second fixation protocol was identical to the first fixation protocol except for the following modifications. Primary fixation was conducted for 28 days instead of overnight. Specimens were dehydrated by sequential incubation in 20%, 40% and 60% EtOH for 10 min at each concentration and 75%, 80%, 85%, 90%, 95% and 100% EtOH for 5 min at each concentration. Specimens sputter coated with 15 nm of gold–palladium. Approximately 37 of 41 embryos were retained with this method, and all were analysed by SEM.

The third fixation protocol was modified from Reed et al. (2021). In brief, primary fixation was conducted in 0.1 M phosphate buffer (pH 7.4) with 3% glutaraldehyde and 3% paraformaldehyde for 24 h at 4°C. To ensure fixative penetration through the cyst wall, specimens were transferred to room temperature (~22°C) for an additional 70 h. After primary fixation, specimens were rinsed twice in 0.1 M phosphate buffer (pH 7.4) for 15 min, followed by an overnight wash in fresh buffer. Secondary fixation was conducted in 0.1 M phosphate buffer (pH 7.4) with 2% osmium tetroxide for 2 h. After secondary fixation, specimens were rinsed twice in 0.1 M phosphate buffer (pH 7.4) for 15 min, followed by an overnight wash in the same buffer. The following morning, specimens were placed in deionized water and kept in deionized water for 24 h. Specimens were then dehydrated by sequential incubation in 30%, 50%, 70% and 95% EtOH for 45 min at each EtOH concentration step before incubating in 100% EtOH for 30 min. Specimens were critically point‐dried with 15 exchanges of liquid carbon dioxide and sputter coated with 13 nm of gold–palladium. Stubs were marked in a quadrant format, and a stereoscope image was taken of each stub to use as a map for tracking embryos for imaging. A total of 192 embryos were retained out of the 200 processed, and all were analysed by SEM. Thus, the majority of embryos visualized in this study used the third fixation method.

Scanning electron micrographs of biofilm present on the visible surface of embryos of *B. poppei* were captured using a Thermo‐FEI Apreo‐S FEG scanning electron microscope (Thermo Fisher Scientific, Waltham, MA, USA) equipped with an external secondary electron detector (Everhart–Thornley detector; ETD). Beam conditions were as follows: 5 kV beam acceleration voltage, 13 pA beam current, 3–5 μs dwell time, 7.5–10 mm working distance, 3072 × 2048 px scan resolution and standard use case. A micrograph of the entire visible surface area of each embryo was taken. Variability in embryo coverage by biofilm was qualitatively assessed using four categories: 0%–10%, 11%–50%, 51%–89% and 90%–100% coverage. Because the embryos are permanently mounted for electron microscopy, only 50% of the surface of each embryo is visible.

### DNA extraction, amplification and concentration

For embryo isolation method #1, microbial DNA was extracted from embryo samples using Qiagen DNeasy® Blood and Tissue Kit (Hilden, Germany) approximately 40.8 ± 10.7 h after isolation of the embryos from the sediment. Embryos were pelleted by low‐speed centrifugation for approximately 15 s using a mini centrifuge (GeneMate BioExpress; VWR International, Radnor, Pennsylvania, USA), and the 0.35‰ AFW supernatant was discarded. Cell lysis was conducted in Qiagen DNeasy® Blood and Tissue Kit Buffer ATL with proteinase K over 4 h at 56°C using an Eppendorf ThermoMixer® F1.5 (Hamburg, Germany) set to 600 rpm. Samples were vortexed for approximately 15 s after lysis and then centrifuged at 2000× gravity for 15 s to pellet cell debris. The supernatant was transferred to a new 1.5 mL centrifuge tube, and DNA purification was conducted according to the manufacturer's instructions. Samples were stored at −20°C and quantified using a NanoDrop® (Thermo Fisher Scientific, Waltham, MA, USA) (Table 1). DNA samples were amplified for the V4 region of the 16S ribosomal subunit to verify microbial DNA was present using universal bacterial/archaeal forward and reverse primers, 515F and 806R, respectively (Caporaso et al., 2011).

### DNA sequencing and data processing

Next‐generation 16S rRNA gene and ITS region DNA sequencing was conducted by Zymo Research Corp., Microbiome Services group (Zymo Research, Irvine, California, USA). Prior to library preparation, a DNA clean‐up step was conducted using the DNA Clean and Concentrator™‐5 (Zymo Research, Irvine, California, USA). Quick‐16S Primer Set V3‐V4 and ZymoBIOMICS Services ITS2 Primer Set were used for library preparation (Zymo Research, Irvine, California, USA). Sequencing of the final library was conducted on an Illumina MiSeq with a v3 reagent kit (600 cycles), and 10% PhiX spike‐in (Supplemental File S1). Data processing was conducted by Zymo Microbiomics. In brief, unique amplicon sequences were inferred from raw reads and chimeric sequences were removed using the DADA2 (Callahan et al., 2019) pipeline. Taxonomy assignment was performed using Uclust (Edgar, 2010) from Qiime v.1.9.1 (Caporaso et al., 2010) and internal Zymo Research Database for reference. Microbial sequences generated in this study were deposited in UNC Dataverse (https://doi.org/10.15139/S3/IENEXB). Updated taxonomic terms based on Panda et al. (2022) were then applied to the taxonomic list generated by the Zymo Research data processing pipeline.

To determine whether it was plausible for one or more microbes found on the surface of the zooplankton embryo to produce chitinolytic proteins, prokaryotic organisms identified by 16S rRNA gene sequencing were searched for on the United States National Center for Biotechnology Information (NCBI) protein and nucleic acid databases. Genera identified in the microbiome were used in combination with the search GH18 chitinase gene family terms: chitinase, glycoside hydrolase family 18 and glycosyl hydrolase family 18. If a search returned a positive result for a GH18 chitinase gene family term in a genus, then an additional NCBI database search was conducted using the species epithet and the same GH18 gene family terms. If the combination of the species name with one of the three chitinase search terms was found, then a single example of a chitinase was provided in Table S2.

## RESULTS

### Microbiota on surface of dormant copepod embryos

**FIGURE 1:**
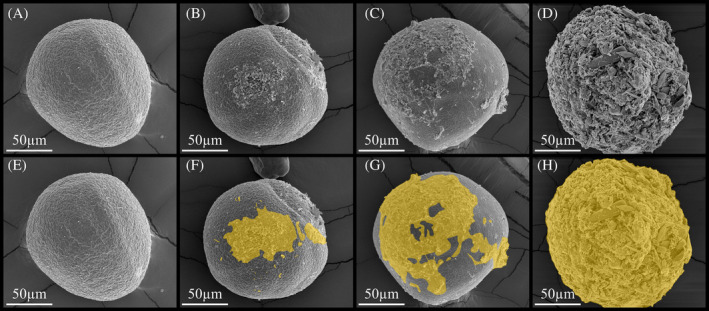
Variability in visible surface area covered by biofilm on encysted dormant embryos of *B. poppei* from stored Antarctic lake sediment, as observed with scanning electron microscopy: (A) 0%–10% biofilm, (B) 11%–50% biofilm, (C) 51%–89% biofilm and (D) 90%–100% biofilm; (E–H) identical to image above with biofilm identified by gold highlight.

When stored in native sediments from King George Island, Antarctica, embryos of the copepod, *B. poppei*, varied greatly in biofilm coverage. In an SEM assessment of 235 embryos, 64.7% of embryos had little biofilm accumulation on their surface (≤10% coverage), and 28.1% of embryos had between 11% and 50% of their cuticular surface covered by biofilm (Figure 1; Table S1). Only 7.2% of embryos had more than 50% of their surface area covered, and only one of those embryos was completely covered by biofilm (Figure 1; Table S1). Vigorous washing of the embryos on a sieve did not decrease the abundance of biofilm on the surface of embryos when compared with gentle rinsing in a cell strainer (Table S1). All embryos used in SEM assessments of biofilm coverage were partially collapsed, and changing the fixation method did not produce spherical embryos.

Differences in the abundance of biofilm on the surface of embryos were observed between two unique sediment grab samples that were fixed with different methods (Table S1). The majority of embryos (77%) isolated with a cell strainer from sediment grab sample #2 had ≤10% biofilm coverage, but only 16% of embryos from sediment grab sample #1 had ≤10% biofilm coverage when isolated with the same method. The majority of embryos (54%) in sediment grab sample #1 were, instead, observed to have 11%–50% coverage with biofilm. By contrast, less than 20% of embryos from sediment grab sample #2 had 11%–50% coverage with biofilm. Only 3% of embryos from sample #2 had >50% coverage with biofilm compared to 30% of embryos from sample #1.

**FIGURE 2:**
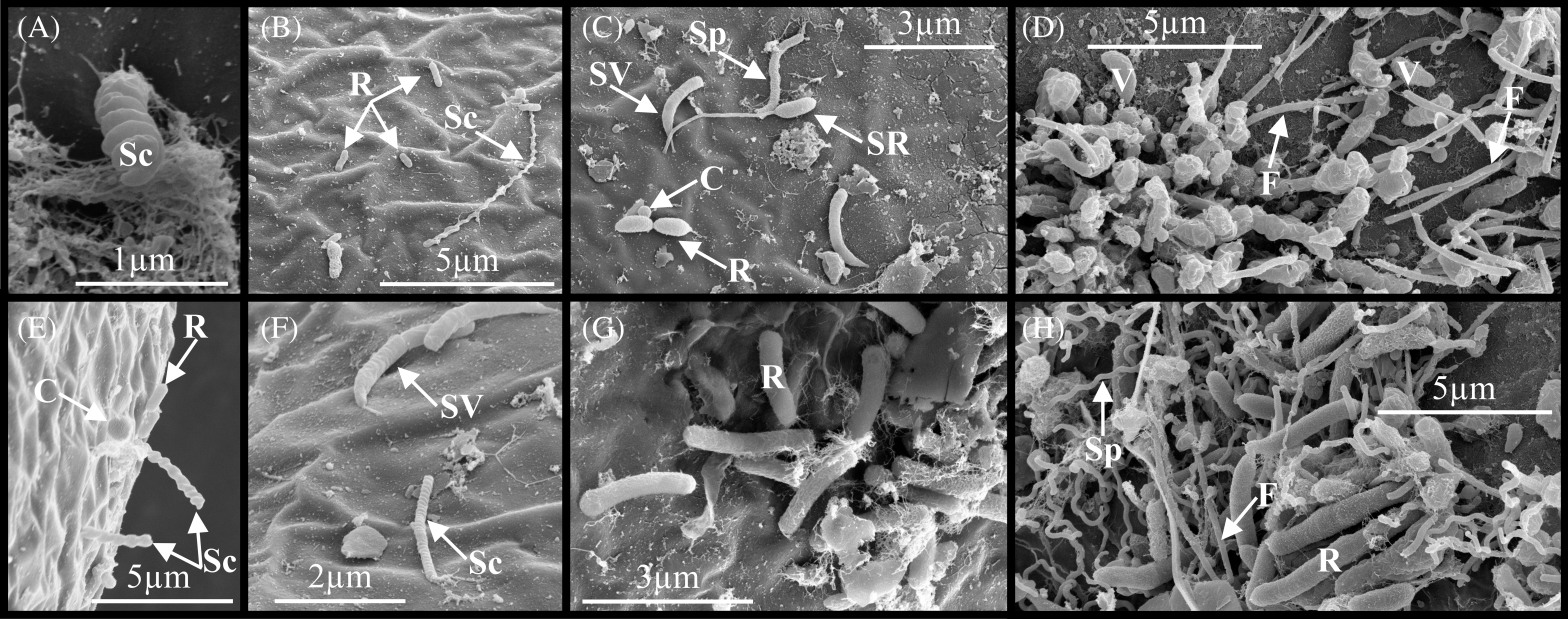
Prokaryotic diversity in biofilm on encysted dormant embryos of the copepod, *B. poppei*, as observed with scanning electron microscopy. Putative identification based on morphology: Spirochete‐shaped (Sc; panels A, B, E and F), rod‐shaped (R; panels B, C, E, G and H), stalked/prosthecate rod‐shaped (SR; panel C), stalked/prosthecate vibrio‐shaped (SV; panels C and F), spirilla‐shaped (Sp; panels C and H), cocci‐shaped (C; panels C and E), filamentous‐shaped (F; panels D and H) and vibrio‐shaped (V; panel D).

Microbes with prokaryotic morphology were observed by SEM in biofilm on the surface of embryos of the copepod, *B. poppei* (Figure 2). Eight distinct categories of prokaryotes were identified based on morphology: spirochete‐shaped, rod‐shaped, rod‐shaped prosthecate (stalked), vibrio‐shaped prosthecate, spirilla‐shaped, cocci‐shaped, filamentous‐shaped and vibrio‐shaped.

**FIGURE 3:**
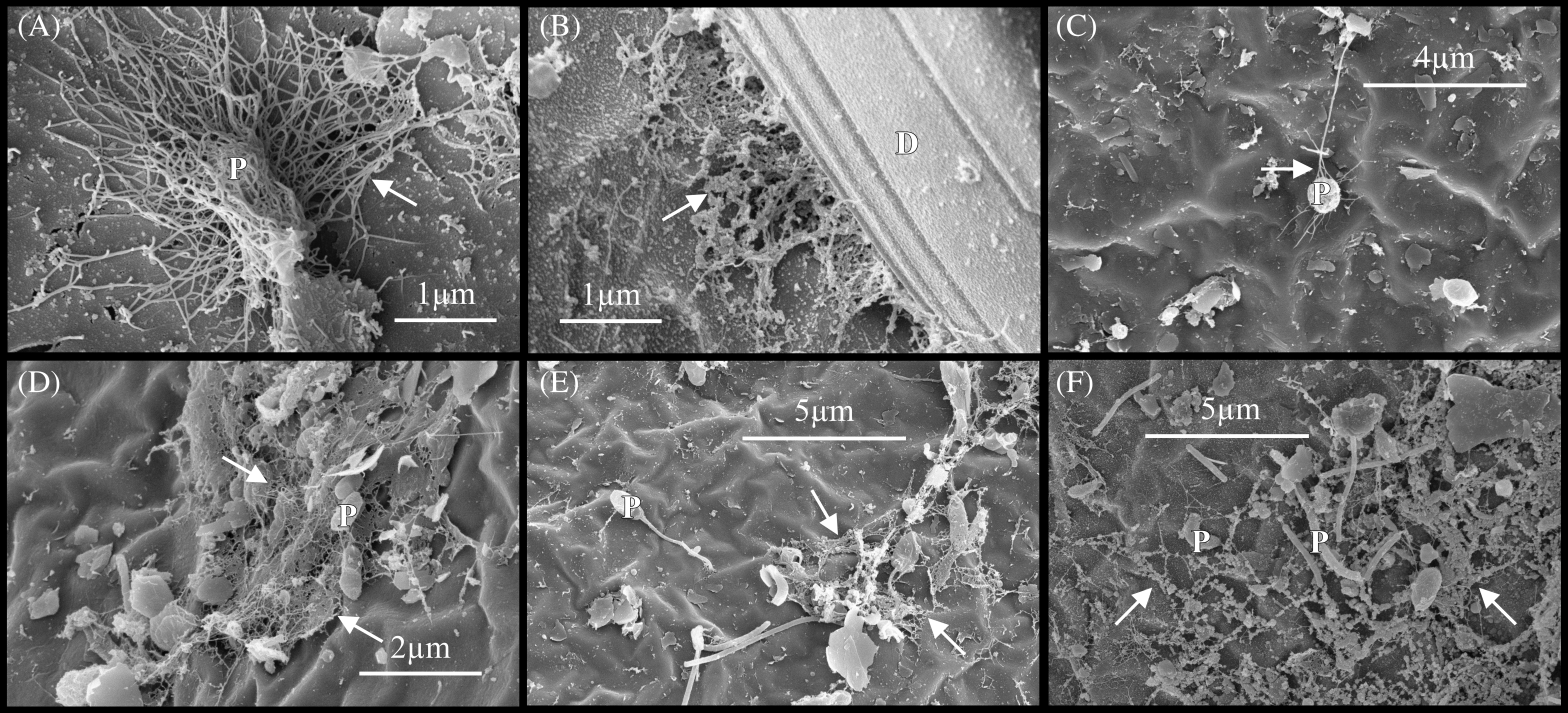
Extracellular polymeric substance (solid white arrows) of putative microbial origin observed in biofilm on the surface of encysted dormant embryos of the copepod, *B. poppei*, using scanning electron microscopy. Extracellular polymeric substance was observed on (A) possible rod‐shaped prokaryote, (B) diatom, (C) cocci‐shaped prokaryote and (D–F) combination of debris and prokaryotes. D, diatom; P, prokaryotes.

**FIGURE 4:**
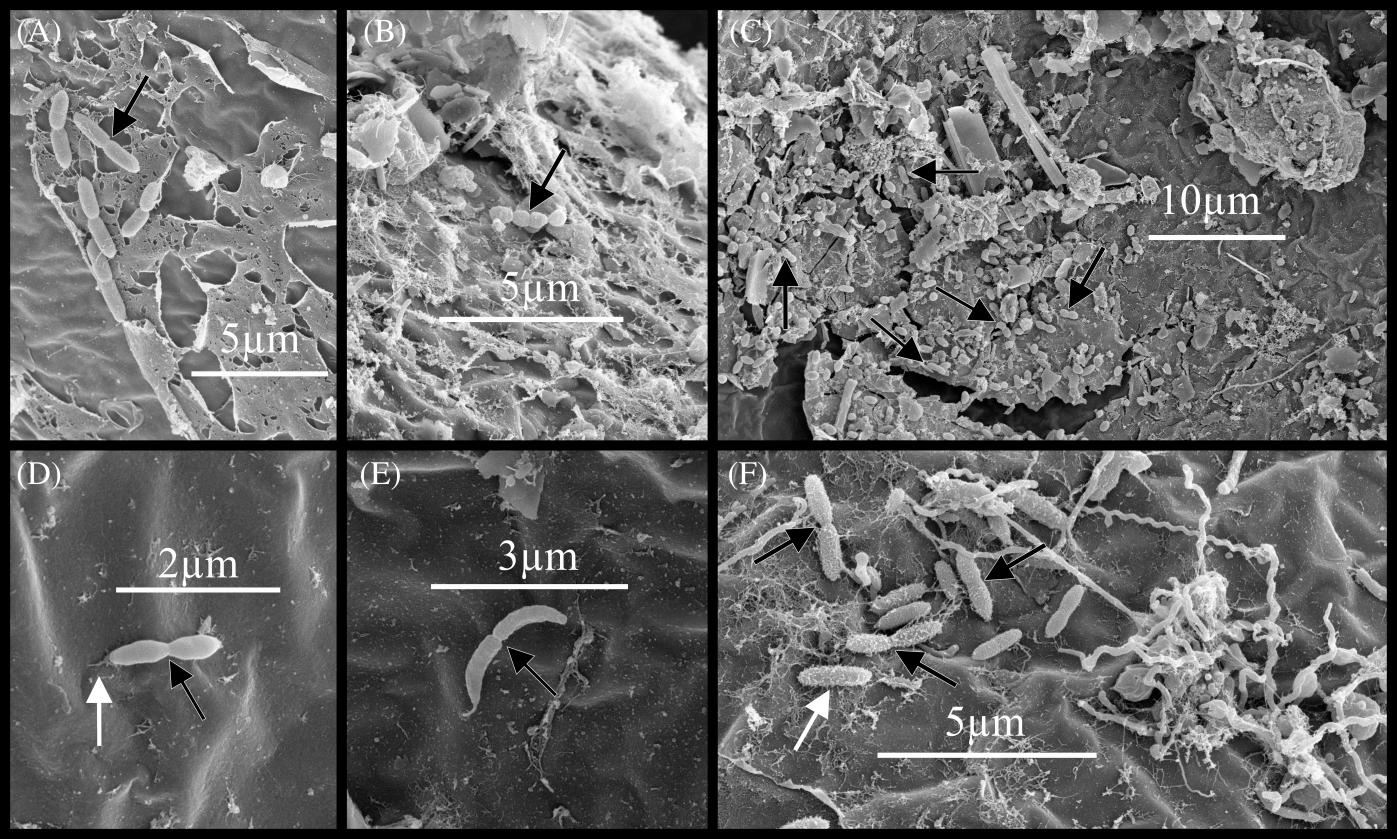
Prokaryotic fission (black arrows) observed in biofilm on the surface of encysted dormant embryos of the copepod, *B. poppei*, using scanning electron microscopy: (A) rod‐shaped prokaryotes, (B) cocci‐shaped prokaryotes, (C) rod‐shaped prokaryotes in complex biofilm, (D) rod‐shaped prokaryote with minor extracellular polymeric substance attachment to embryonic surface, (E) stalked/prosthecate prokaryote with vibrio‐like morphology and (F) rod‐shaped prokaryotes. White arrows indicate extracellular polymeric substance on surface of microbes.

Extracellular polymeric substance (EPS) of putative microbial origin was observed in biofilm on the surface of embryos of the copepod, *B. poppei* (Figure 3). Prokaryotes associated with the EPS had rod‐shaped or cocci‐shaped morphology (Figure 3A,C). Diatoms were sometimes embedded in the EPS (Figure 3B). A net‐like matrix of EPS often surrounded debris and microbial life forming a cohesive microbial mat (Figure 3D–F). Microbes with rod‐shaped, cocci‐shaped and vibrio‐shaped prosthecate prokaryotic morphologies appeared in the process of fission, or asexual reproduction, both within and outside of the microbial mat (Figure 4). EPS was observed on, and as a point of embryonic attachment by, rod‐shaped prokaryotes undergoing asexual reproduction (Figure 4D,F).

**FIGURE 5:**
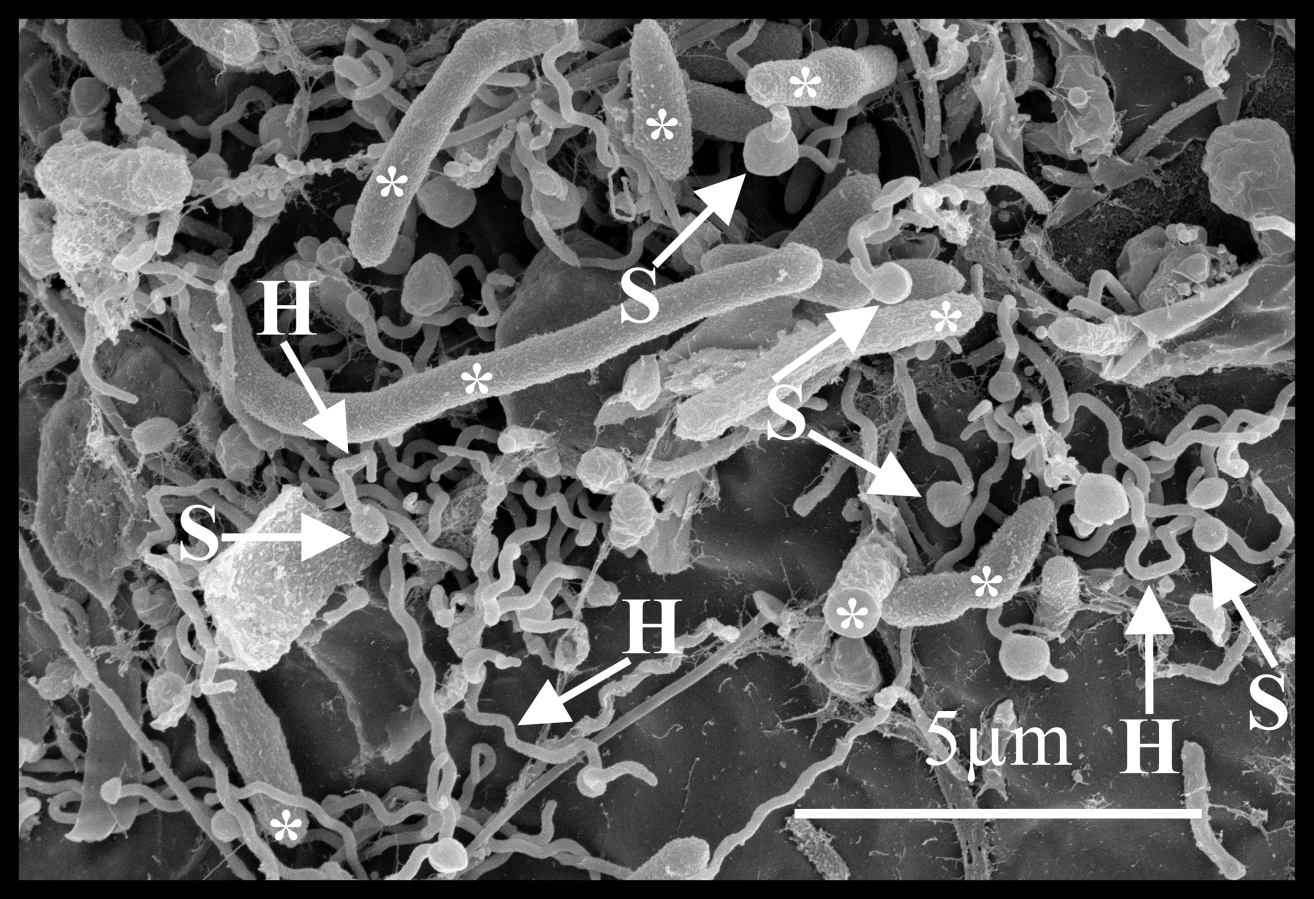
Putative fungal coenocytic hyphae observed in biofilm on the surface of encysted dormant embryos of the copepod, *B. poppei*, using scanning electron microscopy: H, spiral hyphae; S, asymmetrical ovoid spores; *, unidentified microbes.

**FIGURE 6:**
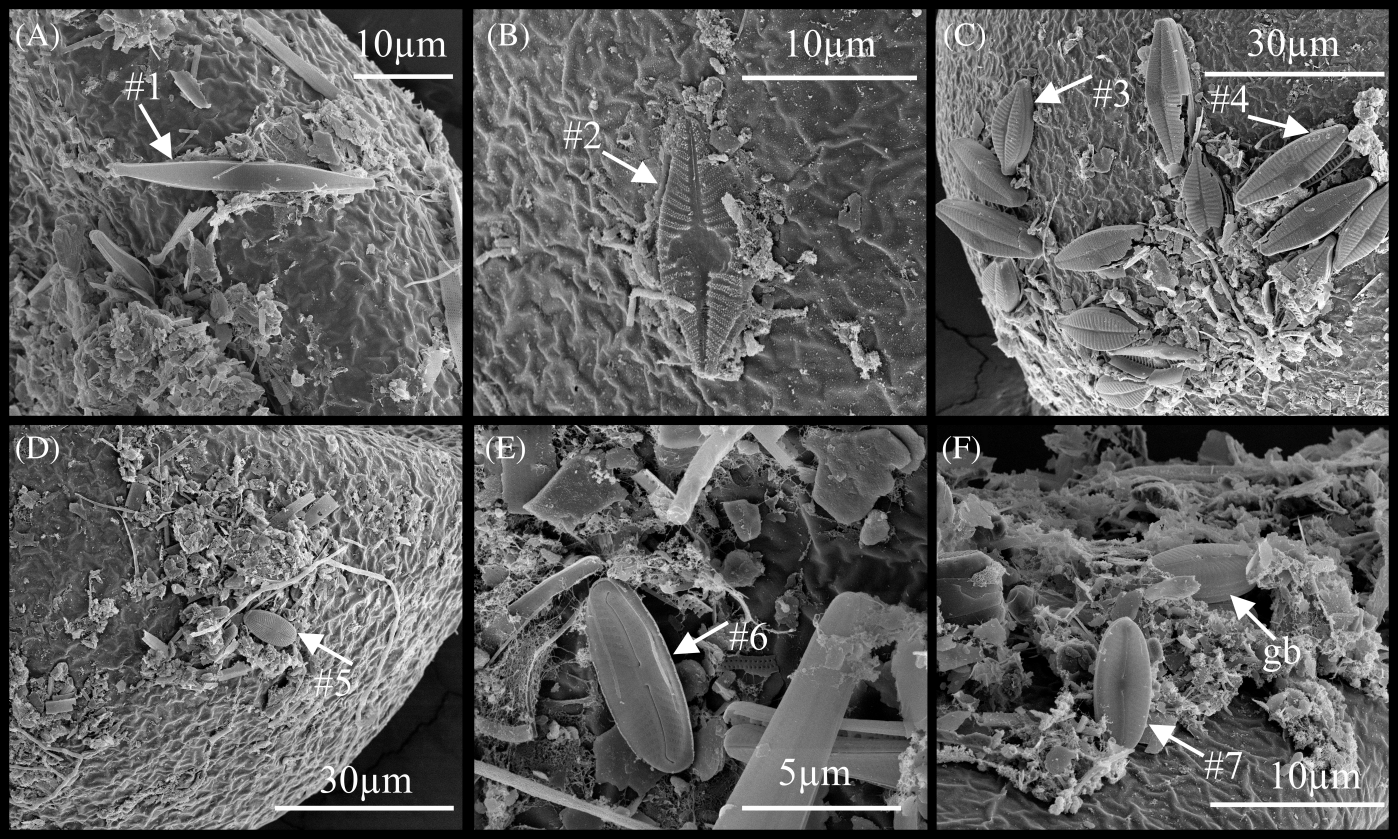
Diatom diversity in biofilm on surface of encysted dormant embryos of the copepod, *B. poppei*, as observed with scanning electron microscopy: (A) araphid pennate diatom; (B–E) raphid pennate diatoms; (F) diatom with visible girdle band (gb). White numbered arrows identify seven potentially unique diatom species or morphotypes.

**FIGURE 7:**
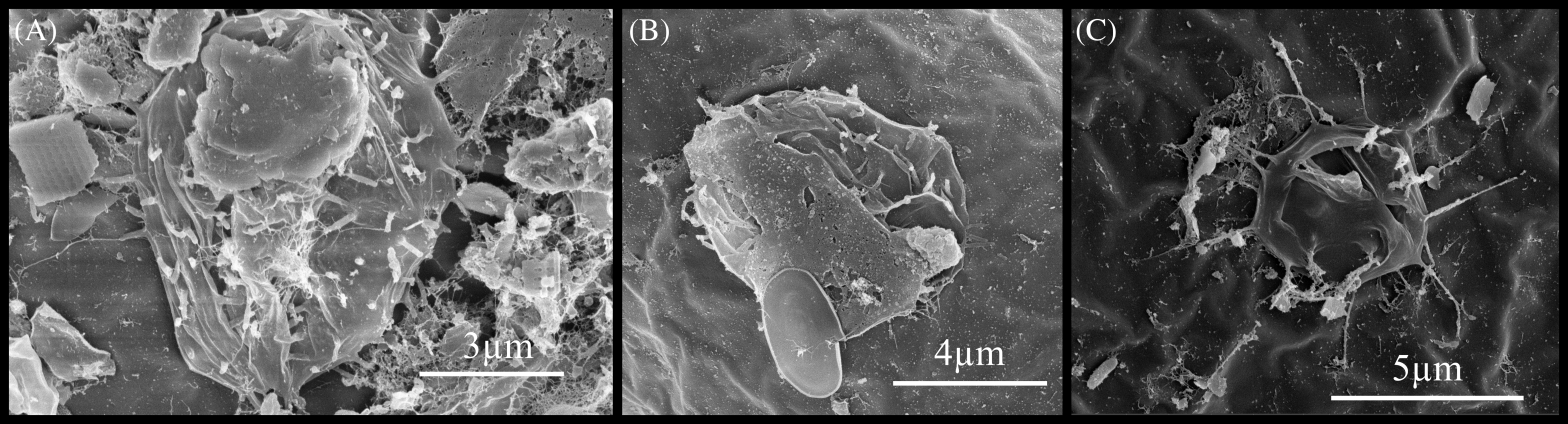
Representative images of amoeboid‐like organisms of varied size in biofilm on surface of encysted dormant embryos of the copepod, *B. poppei*, as observed with scanning electron microscopy. Measured diameter of organisms: (A) 8.2 μm, (B) 7.8 μm and (C) 4.4 μm. Arrows used to identify examples when more than one organism is visible in image.

Eukaryotes were observed in biofilm on the surface of embryos of *B. poppei*, including putative fungi, diatoms and amoeboid organisms (Figures 5 and 6). Fungal hyphae‐like structures with a spiral morphology were observed to extend from ovoid spore‐like structures (Figure 5). Diatom diversity included at least seven different morphotypes (Figure 6). All diatoms observed were pennate in shape, both araphid and raphid (Figure 6). Diatom type #1 was araphid pennate with a pronounced rostrum (Figure 6A). Diatom type #2 was raphid pennate with a defined central area and coarsely spaced radial striae with three rows of puncta per stria (Figure 6B). Diatom type #3 was raphid pennate with a central rib, pronounced rostrum and slightly radial striae with four rows of puncta (Figure 6C). Diatom type #4 was raphid pennate with a central rib, slightly radial striae with three rows of puncta and a more defined central area in comparison to Diatom type #3 (Figure 6C). Both diatom types #3 and #4 had a central area that is shifted to one side of the raphe. Diatom type #5 was raphid pennate with rounded apex regions, defined central area and dense radial rows of striae with a single row of puncta (Figure 6D). Diatom type #6 was raphid pennate with radial striae consisting of a single row of large puncta and a defined central area (Figure 6E). Diatom type #7 was raphid pennate with rounded apex regions and a central area, unclear on puncta and striae (Figure 6F). Girdle bands were also visible on additional diatoms (Figure 6F). Amoeboid organisms were observed less frequently than diatoms or putative fungi and had diameters ranging from 4.4 to 8.2 μm (Figure 7).

Additional unidentified structures of biological origin were observed using scanning electron microscopy. These putative organisms and fragments of organisms had highly varied sizes and were observed with spherical, stalked or hollow tube‐like morphology (Figure S1). The diameter of the hollow tube‐like structures ranged from 1.7 to 0.3 μm (Figure S1).

Microbiome sequencing data using amplification of 16S rRNA gene and ITS region confirmed the presence of prokaryotes and fungi on the surface of dormant copepod embryos (Tables 2 and 3). Archaea and Bacteria were present with a relative abundance of 2.4% and 95.9%, respectively (Table 2). Archaea consisted of a single phylum, Euryarchaeota, which was mostly composed of the class Methanomicrobia (2.3% ± 1.2%). Proteobacteria (now Pseudomonadota), Acidobacteria (now Acidobacteriota), Bacteroidetes (now Bacteroidota), Aminicenantes, Actinobacteria (now Actinomycetota) and Chloroflexi (now Chloroflexota) composed 51.4% ± 10.5%, 9.7% ± 6.9%, 8.6% ± 2.0%, 5.2% ± 3.8%, 3.7% ± 2.0% and 3.3% ± 1.0% of total bacterial abundance, respectively (Table 2). The remaining bacterial community was comprised of 27 phyla that each represented <3% of total abundance. Approximately 1.6% of prokaryotes were unidentified (Table 2). Two fungal phyla were identified: Ascomycota and Basidiomycota (Table 3). Ascomycota is the dominant fungal phylum with a relative abundance of 37.8% ± 2.9%, and Basidiomycota has approximately 0.8% ± 0.1% (Table 3). At least 8.8% ± 5.7% is of unknown fungal phyla and approximately 54.6% ± 12.8% of relative fungal abundance remained unidentified (Table 3).

**TABLE 2:** Prokaryote relative abundance on surface of dormant copepod embryos stored in native Antarctic sediments (data based on 16S ribosomal subunit sequencing; *n* = 5 unique environmental replicates).

Phylum	Class	Mean (%)	SD (%)	Phylum	Class	Mean (%)	SD (%)
Euryarchaeota*	Methanomicrobia	2.3	1.2	Fusobacteria	Fusobacteriia	<0.1	<0.1
	Thermoplasmata	<0.1	<0.1	(Fusobacteriota)			
Acidobacteria	Acidobacteria	0.7	0.2	Gemmatimonadetes	Gemmatimonadetes	0.9	1.0
(Acidobacteriota)	Holophagae	9.0	6.9	(Gemmatimonadota)			
	Acidimicrobiia	1.2	1.2	Gracilibacteria	Unknown	0.2	0.1
Actinobacteria	Actinobacteria	1.4	1.2	Hydrogenedentes	Unknown	0.3	0.5
(Actinomycetota)	Coriobacteriia	0.3	0.2	Latescibacteria	Unknown	1.6	1.2
	Thermoleophilia	0.2	0.2	Lentisphaerae	Lentisphaeria	<0.1	<0.1
	Unknown	0.5	0.4	(Lentisphaerota)	Oligosphaeria	<0.1	<0.1
Aminicenantes	Unknown	5.2	3.8		Unknown	0.1	0.1
Armatimonadetes	Unknown	0.1	0.2	Nitrospirae	Nitrospira	1.4	0.8
(Armatimonadota)				(Nitrospirota)			
Atribacteria	Unknown	<0.1	<0.1	Omnitrophica	Unknown	0.1	0.1
(Atribacterota)				Parcubacteria	Unknown	1.9	1.2
Bacteroidetes	Bacteroidia	0.2	0.1	Planctomycetes	Phycisphaerae	0.2	0.1
(Bacteroidota)	Cytophagia	0.1	<0.1	(Planctomycetota)	Planctomycetacia	1.0	0.7
	Flavobacteriia	0.4	0.4		Unknown	0.2	0.1
	Sphingobacteriia	4.3	1.1	Proteobacteria	Alphaproteobacteria	7.6	7.5
	Unknown	3.6	1.3	(Pseudomonadota)	Betaproteobacteria	9.9	2.7
Caldiserica	Caldisericia	0.1	0.1		Deltaproteobacteria	18.1	13.0
(Calditrichota)					Gammaproteobacteria	15.8	19.9
Chlamydiae	Chlamydiae	0.2	0.2		Unknown	<0.1	<0.1
(Chlamydiota)				Proteobacteria^‡^	Deltaproteobacteria^‡^	0.2	0.1
Chlorobi	Chlorobia	0.1	0.1	(Bdellovibrionota)	(Bdellovibrionales)		
(Chlorobiota)	Ignavibacteria	0.1	<0.1	Proteobacteria^‡^	Epsilonproteobacteria	0.4	0.3
Chloroflexi	Anaerolineae	2.8	1.1	(Campylobacterota)			
(Chloroflexota)	Caldilineae	<0.1	<0.1	Proteobacteria^‡^	Deltaproteobacteria^‡^	0.8	0.6
	Dehalococcoidia	<0.1	<0.1	(Myxococcota)	(Myxococcales)		
	Thermomicrobia	<0.1	<0.1	Saccharibacteria	Unknown	1.0	1.4
	Unknown	0.5	0.4	Spirochaetae	Spirochaetes	0.8	0.5
Cyanobacteria	Chloroplast	0.5	0.4	(Spirochaetota)			
	Cyanobacteria	0.1	0.2	Synergistetes	Synergistia	<0.1	<0.1
	Melainabacteria	<0.1	<0.1	(Synergistota)			
Deferribacteres	Unknown	0.1	0.1	Unknown	Unknown	0.4	0.4
(Deferribacterota)				Verrucomicrobia	Opitutae	0.1	0.1
Elusimicrobia	Elusimicrobia	<0.1	<0.1	(Verrucomicrobiota)	Spartobacteria	<0.1	<0.1
(Elusimicrobiota)					Unknown	0.2	0.1
Firmicutes (Bacillota)	Bacilli	<0.1	<0.1		Verrucomicrobiae	0.3	0.1
	Clostridia	0.8	0.3	Other^†^	Other	1.6	1.3
	Erysipelotrichia	<0.1	<0.1				
	Negativicutes	0.1	<0.1				
	Unknown	<0.1	<0.1				

*Note*: All but one phylum in Kingdom Bacteria.

* Single Archaea phylum identified.

^†^ Unassigned phylum; updated taxonomic terms summarized by Panda et al. (2022) are in parentheses under term provided by Zymo Research.

^‡^ Occurs multiple times in table because original classification was split in the revised taxonomy.

**TABLE 3:** Fungal relative abundance on surface of dormant copepod embryos stored in native Antarctic sediment (based on ITS2 region sequencing; *n* = 5 unique environmental replicates).

Kingdom	Phylum	Class	Order	Family	Genus	Species	Mean (%)	SE (%)
Fungi	Ascomycota	Dothideomycetes	Capnodiales	Cladosporiaceae	Cladosporium	Other	9.4	5.9
					Other	Other	0.4	0.2
			Pleosporales	Phaeosphaeriaceae	Unidentified	Unidentified	0.6	0.6
				Teichosporaceae	Unidentified	Unidentified	0.1	0.1
		Eurotiomycetes	Chaetothyriales	Herpotrichiellaceae	Exophiala	*Exophiala dermatitidis*	0.2	0.2
			Eurotiales	Aspergillaceae	Aspergillus	*Aspergillus conicus*	0.1	0.1
					Penicillium	Other	0.2	0.2
						*Penicillium rubens*	0.7	0.5
					Unidentified	Unidentified	<0.1	<0.1
		Leotiomycetes	Helotiales	Helotiaceae	Meliniomyces	Unidentified	5.0	4.6
				Hyaloscyphaceae	Other	Other	0.2	0.2
				Other	Other	Other	13.9	10.0
				Unidentified	Other	Other	0.8	0.8
					Unidentified	Unidentified	3.4	3.4
		Other	Other	Other	Other	Other	0.3	0.3
		Saccharomycetes	Saccharomycetales	Debaryomycetaceae	Candida	*Candida parapsilosis*	0.3	0.3
				Pichiaceae	Brettanomyces	*Brettanomyces bruxellensis*	<0.1	<0.1
		Sordariomycetes	Coniochaetales	Coniochaetaceae	Other	Other	0.1	0.1
			Hypocreales	Other	Other	Other	0.1	0.1
	Basidiomycota	Malasseziomycetes	Malasseziales	Malasseziaceae	Malassezia	*Malassezia restricta*	0.1	0.1
						Other	0.1	0.1
		Microbotryomycetes	Sporidiobolales	Sporidiobolaceae	Rhodotorula	*Rhodotorula mucilaginosa*	0.4	0.4
		Wallemiomycetes	Wallemiales	Wallemiaceae	Wallemia	Other	0.2	0.2
	Other	Other	Other	Other	Other	Other	8.8	5.7
Unassigned	Other	Other	Other	Other	Other	Other	54.6	12.8

Sequencing of the V3‐V4 region of the 16S rRNA gene resulted in 939 OTUs that fell into 317 genera (Table S2). A total of 106 genera in the microbiome had representatives in the NCBI database with chitinase‐related genes (Table S2). Within these 106 genera, only 29 identified species also had representatives in the NCBI database with chitinase‐related genes (Table S2).

### Novel layers to cyst wall of dormant copepod embryos

**FIGURE 8:**
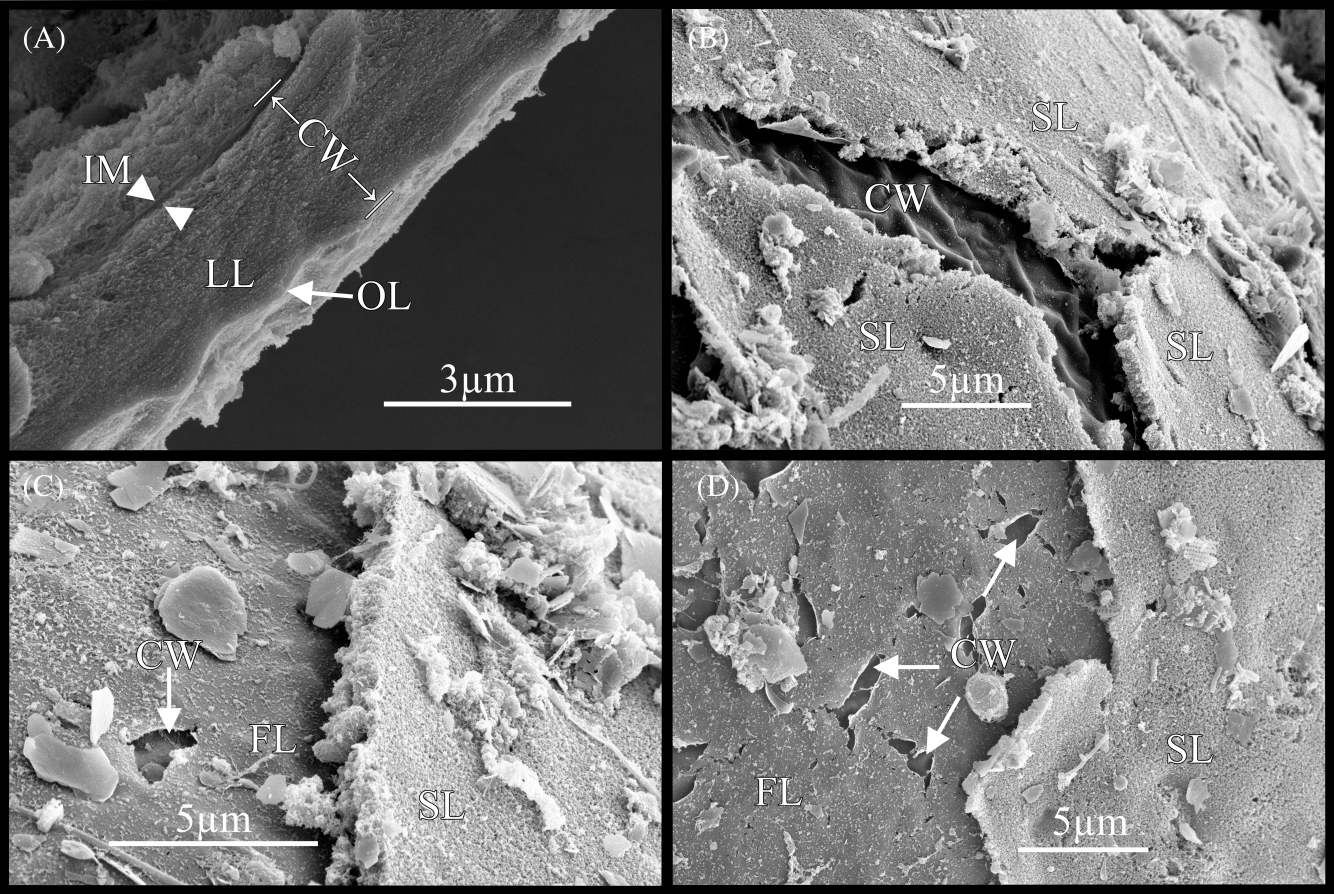
Two previously unidentified layers to the cyst wall in the copepod, *B. poppei*, create a complex environment for microbes on the surface of the embryo. (A) Cross‐section of tri‐layered cyst wall (CW) previously identified by Reed et al. (2021) with thin ‘outer layer’ (OL), a thick lamellar layer (LL), and inner membrane (IM). An additional thin flexible layer (FL) and thicker spongey layer (SL) overlay the previously characterized cyst wall (B–D). Images captured with scanning electron microscopy.

Two additional layers were discovered distal to the tri‐layer cyst wall previously described in the literature (Reed et al., 2021). A cross‐section of the cuticular wall viewed with SEM (Figure 8A) supports the identification of the three cyst‐wall layers described by Reed et al. (2021). However, two additional layers were observed to overlay the ‘outer layer’ described by Reed et al. (2021): a proximal flexible layer and a distal spongey layer (Figure 8). The sponge‐like layer is porous in structure and broke away from the embryo in pieces, but did not fold or twist (Figure 8). By contrast, the thin flexible layer appeared to fold, tear and twist before separating from the embryo (Figure 8; Figure S2). The spongey layer thickness was approximately 0.6–0.8 μm.

## DISCUSSION

It is well accepted that dormant embryos of zooplankton can remain viable for hundreds of years in aquatic sediments (Frisch et al., 2014; Hairston, 1996; Jiang et al., 2012; Marcus et al., 1994), but the mechanisms these dormant embryos use to defend themselves against microbial attack remain unstudied. The present study describes both the embryonic cyst wall and its surface microbiome as a first step toward understanding microbial resistance in dormant embryos of the Antarctic freshwater copepod, *B. poppei*. Two previously undescribed layers of the embryonic cyst wall break apart to provide a complex environment for microbial colonization. By contrast, the third layer remains completely intact with no evidence of damage after years of storage in native Antarctic sediments. Thus, the third layer should be targeted for future investigation of antimicrobial defence. Coverage of the embryos with microbial life varies from sparse population with individual microbes to 100% coverage by a thick biofilm (Figure 1), but most embryos have <50% coverage with biofilm (Table S1). It is currently unclear if the third cuticular layer makes it difficult for microbes to colonize the surface or if it recruits beneficial microbes.

To the best of the authors' knowledge, this study is the first to evaluate the microbial community colonizing the surface of dormant benthic organisms in Antarctic freshwater lakes. Observations with SEM demonstrate that prokaryotes are the dominant organisms colonizing the surface of copepod embryos stored in native Antarctic sediments (Figure 2). A microbiome analysis supports SEM observations and demonstrates the presence of at least 33 prokaryotic phyla (Table 2). Proteobacteria (now Pseudomonadota) are the dominant bacterial phylum in maritime freshwater lakes of the Antarctic (Rochera & Camacho, 2019), and they are also the dominant phylum on the surface of dormant copepod embryos (Table 2). In total, 51.4% of prokaryotic relative read abundance consists of Proteobacteria (now Pseudomonadota). The next most common phyla are Acidobacteria (now Acidobacteriota) and Bacteroidetes (now Bacteroidota) at 9.7% and 8.6% of total relative read abundance, respectively. Only two classes of Archaea were detected on the surface of the dormant copepods, and the Archaea represent 2.4% of total prokaryotic read abundance (Table 2).

It is unknown how dormant zooplankton defend themselves against cold‐adapted microbes that produce chitinases. At least two species of cold‐adapted bacteria (*Pseudomonas* strain GWSMS‐1 and *Sanguibacter antarcticus*) that are known to produce chitinases (Lee et al., 2010; Liu et al., 2019) were not detected on the surface of dormant *B. poppei* (Table S2). However, 29 other OTUs that were identified to species have recorded chitinase genes in the NCBI database which are present on the surface of the embryos. Other zooplankton, such as *Daphnia*, are known to have bacterial epibionts (Eckert & Pernthaler, 2014). Such epibionts could outcompete potentially pathogenic species that produce chitinases. Further study is needed to evaluate whether the surface of dormant embryos in Antarctic lake sediments promote the development of a microbiome that selects against potentially pathogenic microbes.

Both sequencing of the ITS region and visualization with SEM demonstrate the presence of fungi on the surface of dormant copepod embryos stored in native sediments from Antarctic lakes (Figures 1 and 5). Fungi associate with sessile marine invertebrates (Yarden, 2014), so it is not surprising to find them on the surface of dormant embryos in bottom sediments of maritime lakes. Freshwater fungi are well documented in Antarctic lakes (de Souza et al., 2021; Doytchinov & Dimov, 2022; Hatano et al., 2022; Rosa et al., 2022). Cold‐adapted yeasts are also present in Antarctica (Baeza et al., 2022). Ascomycota and Basidiomycota are the dominant culturable fungal phyla in a lake on King George Island, Antarctica, accounting for 96% of isolates (de Souza et al., 2023). This dominance pattern is consistent with ITS sequencing data for the identifiable fungi on the surface of dormant copepod embryos from another lake on King George Island (Table 3). However, 55% ± 12% of fungal abundance on copepod embryos remain unidentified (Table 3). Because Oomycetes and fungi are among the few microbes capable of penetrating crustacean cuticle (Coates et al., 2022), it is critical to document the fungi that colonize dormant copepods. To the best of the authors' knowledge, none of the fungi identified in the present study cause crustacean shell disease.

After up to 3.3 years of storage in sealed containers at 4°C, a portion of the microbial community remains viable. The presence of bacterial fission in the microbial community on the surface of stored dormant copepod embryos suggests that some prokaryotes are viable (Figure 4). Amoeboid organisms also appear viable on the embryo surface (Figure 7), because they lack hardened structures that would persist after death. By contrast, it is not possible to know if diatoms trapped on the surface of copepod embryos are viable. The presence of diatoms is expected because diatoms are found in the water column and benthic environments of Antarctic freshwater lakes (Bertoglio et al., 2023; Camara et al., 2021; Prelle et al., 2022; Zebek et al., 2021; Zhang et al., 2022). Viability is also theoretically possible due to ‘dark metabolism’, where only essential metabolic pathways are sustained in the dark (Kennedy et al., 2019). Embryos and microbes must experience dark oxygen limiting conditions when lakes are frozen over during the austral winter, and the duration of storage in the present study is a minor fraction of the ~200 years this species is capable of (Jiang et al., 2012).

Microbes identified on the surface of the Antarctic copepod embryos used in this study originate from the Antarctic lake where the embryos were collected, but antimicrobial defences must work in diverse environments with diverse microbial communities. All practicable steps were taken to seal samples in Antarctica and prevent the introduction of non‐native microbes during storage and processing for microscopy and DNA isolation. That said, it is reasonable to assume that the microbial community changed during shipping and storage. Importantly, all studies on zooplankton embryos require storage of embryos in native sediments for many months. A comparison of embryo abundance at the time of collection to the date of processing for experiments demonstrates little or no change in embryo abundance during storage when sediment subsamples are not opened (Reed et al., 2021). This prolonged storage, and the prolonged survival in nature, demonstrate that most of those embryos successfully defend against microbial attack despite changes in the microbial community. Furthermore, zooplankton species like *B. poppei* have broad geographic distributions (Maturana et al., 2019), across which a broad diversity of microbial communities exist. Thus, passive antimicrobial defences in dormant embryos of copepods like *B. poppei* must work against a broad diversity of microbes.

A previous study demonstrated the presence of three cyst wall layers in the Antarctic copepod, *B. poppei* (Reed et al., 2021), but the present study confirms the presence of five layers. Scanning electron micrographs (Figure 8) reveal an overlaying thin cuticle‐like fourth layer just outside of the third layer described by Reed et al. (2021). A distal‐most sponge‐like fifth layer forms the true outer layer in diapause embryos of *B. poppei*, and is 0.6–0.8 μm thick (Figure 8). These two newly identified layers were previously described as a single ‘egg membrane’ that was only present in younger diapause embryos of *Boeckella triarticulata* (Couch et al., 2001). However, a comparison of the micrographs in the present study with this previous study on *B. triarticulata* demonstrates that the ‘egg membrane’ consists of two layers in *Boeckella* species. Both layers break away from the embryo and leave behind the three‐layered cyst wall observed in previous studies on *B. poppei*. A complex microbial community is present on the third cyst‐wall layer, indicating that loss of the outer layers occurs in the native environment or during storage in native sediments (Figures 1, 2, 3, 4, 5, 6, 7). Assessment of the degradative process is beyond the scope of the present study. However, it is worth noting that no erosion or pitting of this third layer was observed in any the 235 embryos examined for this study. The lack of damage to the third cyst wall layer suggests that it is either resistant to enzymatic degradation or is protected from chitinase producing microbes by beneficial microbes.

The thin flexible layer (fourth layer) of the cyst wall described in this study is similar in structure under SEM to the cuticular hatching membrane observed in brine shrimp, which is composed of chitin. Tearing, folding and stretching of the thin layer indicates that it is flexible (Figure 8; Figure S2). The most extensive developmental study on a zooplankton ever published indicates that diapause embryos of the brine shrimp may undergo the first moult cycle in utero and that the embryonic cuticle observed in diapause embryos is actually the second embryonic cuticle (Benesch, 1969). Thus, it is possible that the thin flexible layer is the first embryonic cuticle. A test of this hypothesis would require observations of development through the gastrula‐stage in utero.

A comparison with diapause embryos of the brine shrimp suggests that the distal‐most sponge‐like layer of the cyst wall in diapause embryos of *B. poppei* is maternal in origin and not critical to protection of the embryo. The breakage pattern observed in SEM images suggests that this layer is rigid (Figure 8). The same texture and breakage pattern is apparent in an outer‐most layer that is produced by a maternal shell gland in dormant embryos of the brine shrimp, *A. franciscana* (Anderson et al., 1970). A key difference is that the outer two layers in *B. poppei* breaks away over time while the maternal proteinaceous chorion of brine shrimp embryos remains intact until hatching, and acts as a barrier to lipophilic chemicals (Covi & Hand, 2005). A comparison of the composition of these outermost layers among species may reveal why the layer is resistant to breakage in *Artemia*.

Extracellular polymeric substance is present on the surface of dormant *B. poppei*, and its importance should not be overlooked. Microbes use EPS to create a biofilm for cryoprotection, desiccation protection, and the sequestration of nutrients and organic matter (Nagar et al., 2021). Thus, the EPS could increase survivorship in the biofilm during long‐term storage in the lab or burial in nature. The EPS also holds fragments of the outer embryo cyst wall in place with the biofilm (Figure 3D) and creates a complex topology for colonization by microbes (Figure 1C,D).

## CONCLUSION

This is the first study to investigate the composition of the microbial community colonizing the surface of dormant embryos of the Antarctic freshwater copepod, *Boeckella poppei*. Evaluation of the embryo surface with SEM reveals a highly variable environment with some archaeans and a high diversity of bacteria and fungi. The flaking of two previously undescribed layers of the embryo cuticle increases the complexity of the embryo surface and may promote community diversity. The lack of damage to the third layer of the cyst wall indicates that it serves as the primary barrier against microbial attack. Future research should investigate the nature of the third cuticular layer to determine how it allows microbes to colonize the embryo surface without causing damage. Additionally, research is needed to determine whether the microbial community on the surface of dormant zooplankton embryos differs substantially from the sediment in which these embryos reside for years to centuries.

## AUTHOR CONTRIBUTIONS

**Hunter B. Arrington:** Conceptualization (equal); data curation (equal); formal analysis (equal); investigation (equal); methodology (equal); software (lead); validation (equal); visualization (equal); writing – original draft (equal); writing – review and editing (equal). **Sung Gu Lee:** Formal analysis (supporting); funding acquisition (equal); investigation (supporting); project administration (supporting); resources (equal); supervision (supporting); writing – review and editing (equal). **Jun Hyuck Lee:** Formal analysis (supporting); funding acquisition (equal); investigation (supporting); project administration (supporting); resources (equal); supervision (supporting); writing – review and editing (equal). **Joseph Covi:** Conceptualization (equal); data curation (equal); formal analysis (equal); funding acquisition (equal); investigation (equal); methodology (equal); project administration (equal); resources (equal); software (supporting); supervision (equal); validation (equal); visualization (equal); writing – original draft (equal); writing – review and editing (equal).

## CONFLICT OF INTEREST STATEMENT

The authors declare no conflicts of interest.

## Supporting information


**Data S1.** Supporting information.

**Figure S1.** Unidentified organisms in biofilm on surface of encysted dormant embryos of the copepod, *B. poppei*, as observed with scanning electron microscopy. (A) flattened polymorphic with extracellular polymeric substance, (B–F) spherical with textured surfaces, (G) spherical with intricate folded surface, (H) spherical with coccolithophore‐like disks on surface, (I) spherical with hexagon ridges on surface, (J) large organism with stalk, (K, L) small disks with long stalked structures adhering to embryo surface, and tube‐like structures of varied size: (M) 1.7 μm diameter and (N) 0.3 μm diameter. Arrows identify representative examples.

**Figure S2.** Additional flexible layer (FL) observed between the cyst wall (CW) ‘outer layer’ and spongey layer folds, tears and twists (arrows). *, unidentified microbes.

**Table S1.** Summary of isolation and fixation methods for *B. poppei* used in assessments of biofilm coverage on surface of dormant embryos.

**Table S2.** Prokaryotic OTUs identified to species from 16S rRNA gene sequencing using Zymo Research Internal Database for initial OTU identification; NCBI protein and nucleic acid databases were searched using a combination of each genus name with the GH18 chitinase gene family terms: chitinase, glycoside hydrolase family 18, and glycosyl hydrolase family 18; ‘Yes’ indicates a positive identification of one or more of these genes with that genus. If a positive identification was found at the genus level, then a second search was conducted at the species level with the same gene terms. This method provide 106 genera and 29 species with NCBI representatives; links to sources are provided below.

## Data Availability

The raw data for the microbiome are openly available on UNC Dataverse at https://doi.org/10.15139/S3/IENEXB.
