# Supplementary material for: Assessment of the cyst wall and surface microbiota in dormant embryos of the Antarctic calanoid copepod, Boeckella poppei
Source: Environ Microbiol Rep. 2024 Nov 27;16(6):e70035. doi: 10.1111/1758-2229.70035 (PMC11602222; doi:10.1111/1758-2229.70035)
Supplement: Supplementary file 1 — Data S1. Supporting information. [file EMI4-16-e70035-s003.pdf]

 Pages not specific to methodology were deleted

ZYMO RESEARCH

Microbiome Sequencing Service Report:
16S & ITS Amplicon Sequencing

Workflow Checklist

Sample Received	✓
Sample Quality Evaluated	✓
Sample Prepared for Sequencing	✓
Next-Gen Sequencing	✓
Sequence Quality Check	✓
Bioinformatics Processing	✓
Absolute Abundance	✓
Data/Results	✓

ZYMO RESEARCH CORP.
Phone: (949) 679-1190 ▪ Toll Free: (888) 882-9682 ▪ Fax: (949) 266-9452
services@zymoresearch.com ▪ info@zymoresearch.com ▪ www.zymoresearch.com

ZYMO RESEARCH

Methods

The samples were processed and analyzed with the ZymoBIOMICS® Service: Targeted Metagenomic Sequencing (Zymo Research, Irvine, CA).

DNA Extraction: If DNA extraction was performed, one of three different DNA extraction kits was used depending on the sample type and sample volume and were used according to the manufacturer's instructions, unless otherwise stated. The kit used in this project is marked below.

☐ ZymoBIOMICS® DNA Miniprep Kit (Zymo Research, Irvine, CA)

☐ ZymoBIOMICS® DNA Microprep Kit (Zymo Research, Irvine, CA)

☒ ZymoBIOMICS®-96 MagBead DNA Kit (Zymo Research, Irvine, CA)

☐ N/A (DNA Extraction Not Performed)

Elution Volume: 50µL

Additional Notes: The DNA Clean & Concentrator™-5 (Zymo Research, Irvine, CA) was used to purify DNA prior to library preparation

Targeted Library Preparation: The DNA samples were prepared for targeted sequencing with the *Quick*-16S™ NGS Library Prep Kit (Zymo Research, Irvine, CA). These primers were custom-designed by Zymo Research to provide the best coverage while maintaining high sensitivity. The primer sets used in this project are marked below.

☐ *Quick*-16S™ Primer Set V1-V2 (Zymo Research, Irvine, CA)

☐ *Quick*-16S™ Primer Set V1-V3 (Zymo Research, Irvine, CA)

☒ *Quick*-16S™ Primer Set V3-V4 (Zymo Research, Irvine, CA)

☐ *Quick*-16S™ Primer Set V4 (Zymo Research, Irvine, CA)

☐ *Quick*-16S™ Primer Set V6-V8 (Zymo Research, Irvine, CA)

☒ ZymoBIOMICS® Services ITS2 Primer Set (Zymo Research, Irvine, CA)

☐ Other:

Additional Notes: NA

The sequencing library was prepared using an innovative library preparation process in which PCR reactions were performed in real-time PCR machines to control cycles and therefore limit PCR chimera formation. The final PCR products were quantified with qPCR fluorescence readings and pooled together based on equal molarity. The final pooled library was cleaned up with the Select-a-Size DNA Clean & Concentrator™

ZYMO RESEARCH CORP.
Phone: (949) 679-1190 ▪ Toll Free: (888) 882-9682 ▪ Fax: (949) 266-9452
services@zymoresearch.com ▪ info@zymoresearch.com ▪ www.zymoresearch.com

ZYMO RESEARCH

(Zymo Research, Irvine, CA), then quantified with TapeStation® (Agilent Technologies, Santa Clara, CA) and Qubit® (Thermo Fisher Scientific, Waltham, WA).

ZYMO RESEARCH CORP.
Phone: (949) 679-1190 ▪ Toll Free: (888) 882-9682 ▪ Fax: (949) 266-9452
services@zymoresearch.com ▪ info@zymoresearch.com ▪ www.zymoresearch.com

ZYMO RESEARCH

Methods (Cont.)

Control Samples: The ZymoBIOMICS® Microbial Community Standard (Zymo Research, Irvine, CA) was used as a positive control for each DNA extraction, if performed. The ZymoBIOMICS® Microbial Community DNA Standard (Zymo Research, Irvine, CA) was used as a positive control for each targeted library preparation. Negative controls (i.e. blank extraction control, blank library preparation control) were included to assess the level of bioburden carried by the wet-lab process.

Sequencing: The final library was sequenced on Illumina® MiSeq™ with a v3 reagent kit (600 cycles). The sequencing was performed with 10% PhiX spike-in.

Bioinformatics Analysis: Unique amplicon sequences were inferred from raw reads using the Dada2 pipeline (Callahan et al., 2016). Chimeric sequences were also removed with the Dada2 pipeline. Taxonomy assignment was performed using Uclust from Qiime v.1.9.1. Taxonomy was assigned with the Zymo Research Database, a 16S database that is internally designed and curated, as reference.

Composition visualization, alpha-diversity, and beta-diversity analyses were performed with Qiime v.1.9.1 (Caporaso et al., 2010). If applicable, taxonomy that have significant abundance among different groups were identified by LEfSe (Segata et al., 2011) using default settings. Other analyses such as heatmaps, Taxa2SV_deomposer, and PCoA plots were performed with internal scripts.

Absolute Abundance Quantification*: A quantitative real-time PCR was set up with a standard curve. The standard curve was made with plasmid DNA containing one copy of the 16S gene and one copy of the fungal ITS2 region prepared in 10-fold serial dilutions. The primers used were the same as those used in Targeted Library Preparation. The equation generated by the plasmid DNA standard curve was used to calculate the number of gene copies in the reaction for each sample. The PCR input volume (2 µl) was used to calculate the number of gene copies per microliter in each DNA sample.

The number of genome copies per microliter DNA sample was calculated by dividing the gene copy number by an assumed number of gene copies per genome. The value used for 16S copies per genome is 4. The value used for ITS copies per genome is 200. The amount of DNA per microliter DNA sample was calculated using an assumed genome size of 4.64×10^6 bp, the genome size of *Escherichia coli*, for 16S samples, or an assumed genome size of 1.20×10^7 bp, the genome size of *Saccharomyces cerevisiae*, for ITS samples. This calculation is shown below:

Calculated Total DNA = Calculated Total Genome Copies × Assumed Genome Size (4.64×10^6 bp) × Average Molecular Weight of a DNA bp (660 g/mole/bp) ÷ Avogadro's Number (6.022×10^{23}/mole)

The absolute abundance standard curves are shown on page 5 of this report.

*Absolute Abundance Quantification is only available for 16S and ITS analyses.

ZYMO RESEARCH CORP.
Phone: (949) 679-1190 ▪ Toll Free: (888) 882-9682 ▪ Fax: (949) 266-9452
services@zymoresearch.com ▪ info@zymoresearch.com ▪ www.zymoresearch.com

Absolute Abundance Standard Curves

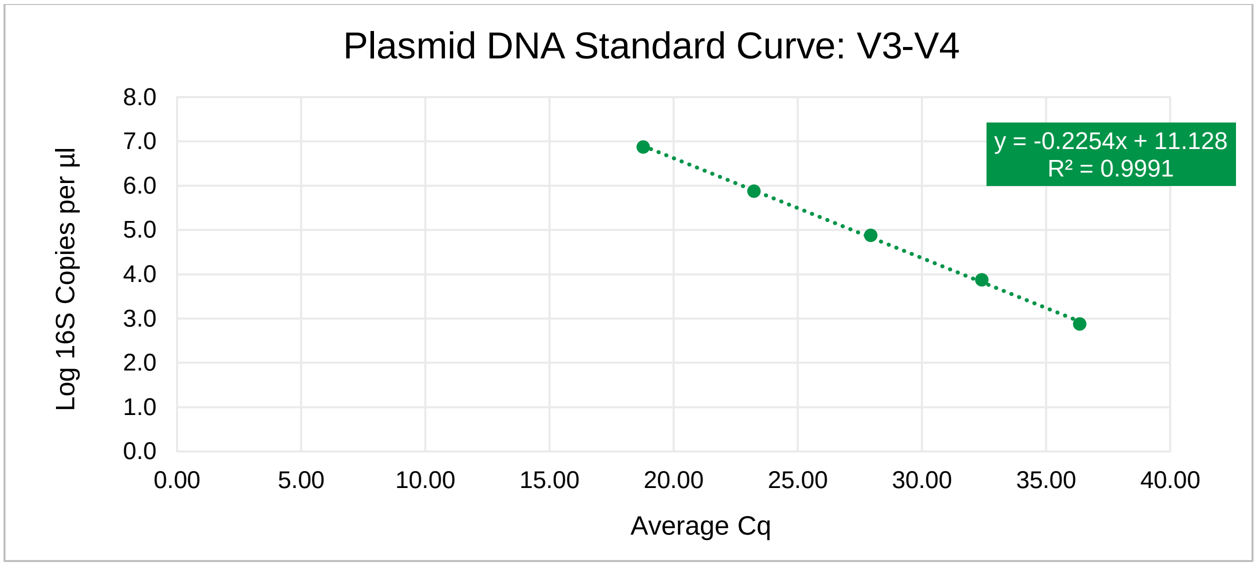

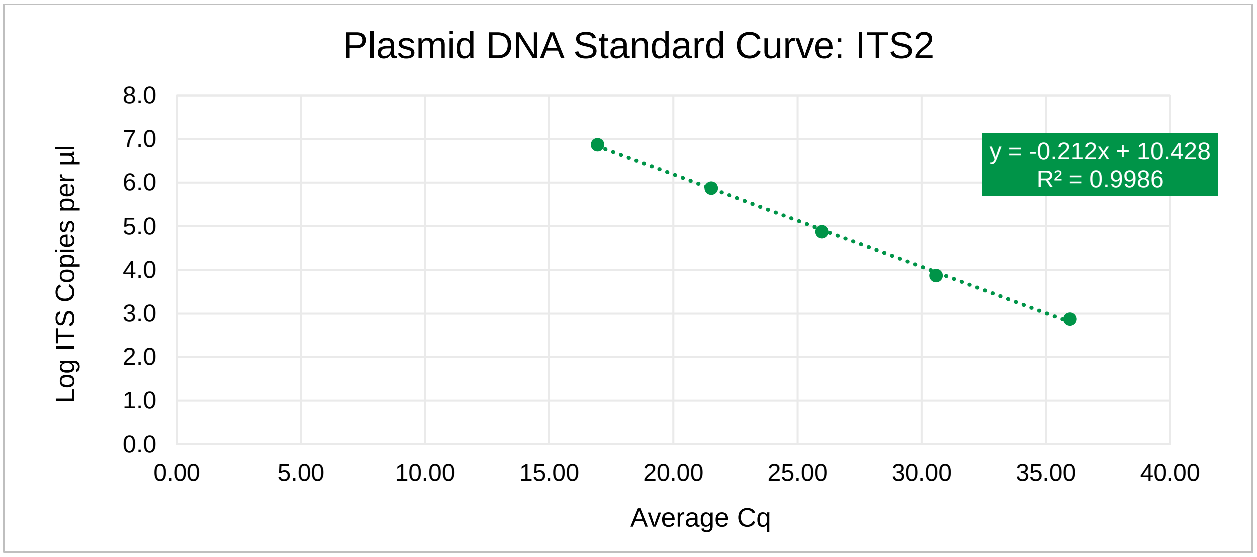

ZYMO RESEARCH CORP.
Phone: (949) 679-1190 ▪ Toll Free: (888) 882-9682 ▪ Fax: (949) 266-9452
services@zymoresearch.com ▪ info@zymoresearch.com ▪ www.zymoresearch.com

ZYMO RESEARCH

References

Callahan, B.J., McMurdie, P.J., Rosen, M.J., Han, A.W., Johnson, A.J., Holmes, S.P. (2016). DADA2: High resolution sample inference from Illumina amplicon data. Nat Methods 13(7):581-3. doi: 10.1038/nmeth.3869

Caporaso, J.G., Kuczynski, J., Stombaugh, J., Bittinger, K., Bushman, F.D., Costello, E.K. ... Knight R. (2010). QIIME allows analysis of high-throughput community sequencing data. Nat Methods (7): 335-336. doi: 10.1038/nmeth.f.303

Segata, N., Izard, J., Waldron, L., Gevers, D., Miropolsky, L., Garrett, W.S., and Huttenhower, C. (2011) Metagenomic biomarker discovery and explanation. Genome Biol (12): R60. doi: 10.1186/gb-2011-12-6-r60
