# Supplementary figures and images for: Assessment of the cyst wall and surface microbiota in dormant embryos of the Antarctic calanoid copepod, Boeckella poppei
Source: Environ Microbiol Rep. 2024 Nov 27;16(6):e70035. doi: 10.1111/1758-2229.70035 (PMC11602222; doi:10.1111/1758-2229.70035)

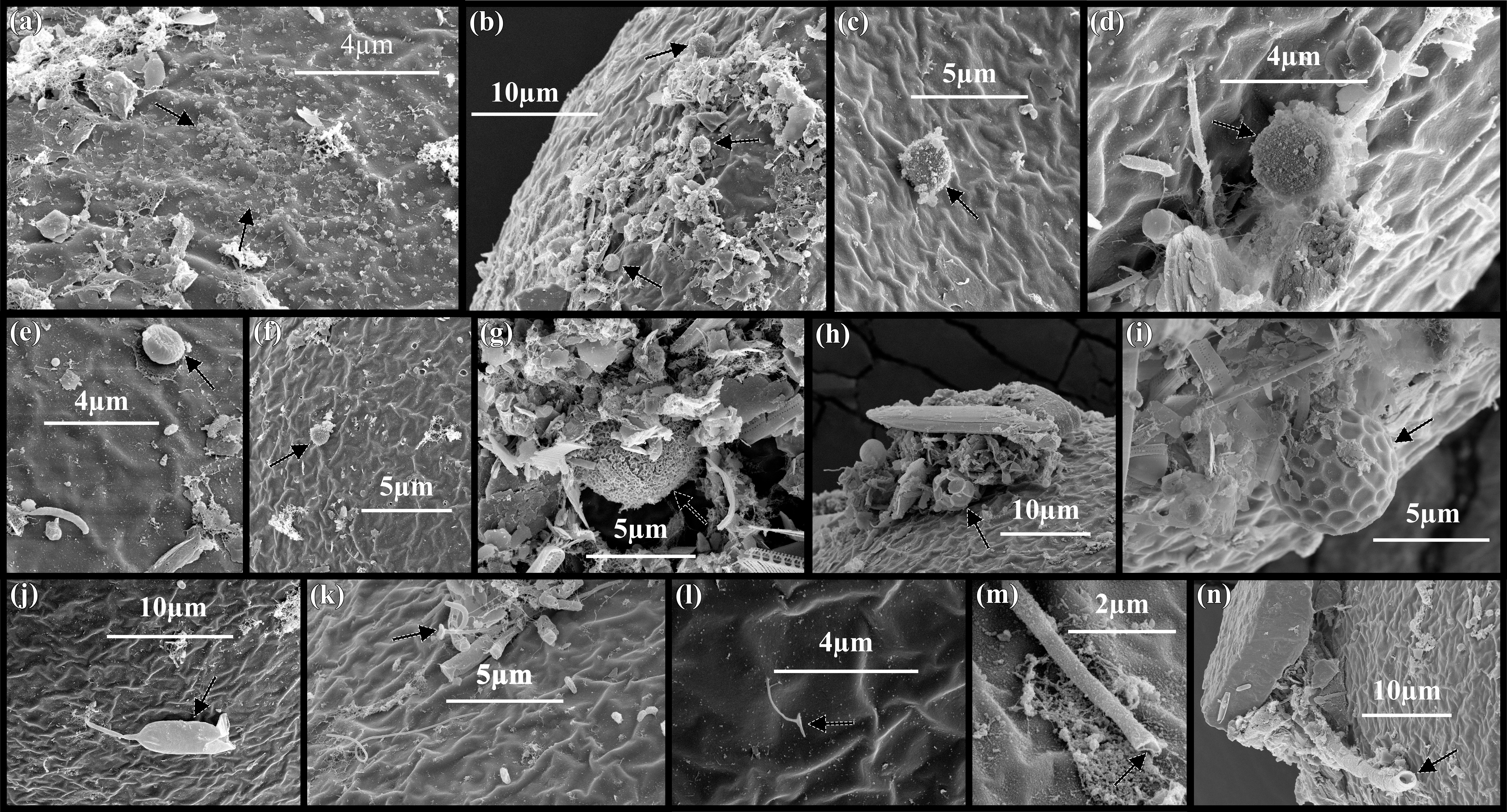

Supplement: Supplementary file 2 — Figure S1. Unidentified organisms in biofilm on surface of encysted dormant embryos of the copepod, B. poppei, as observed with scanning electron microscopy. (A) flattened polymorphic with extracellular polymeric substance, (B–F) spherical with textured surfaces, (G) spherical with intricate folded surface, (H) spherical with coccolithophore‐like disks on surface, (I) spherical with hexagon ridges on surface, (J) large organism with stalk, (K, L) small disks with long stalked structures adhering to embryo surface, and tube‐like structures of varied size: (M) 1.7 μm diameter and (N) 0.3 μm diameter. Arrows identify representative examples. [file EMI4-16-e70035-s002.tif]

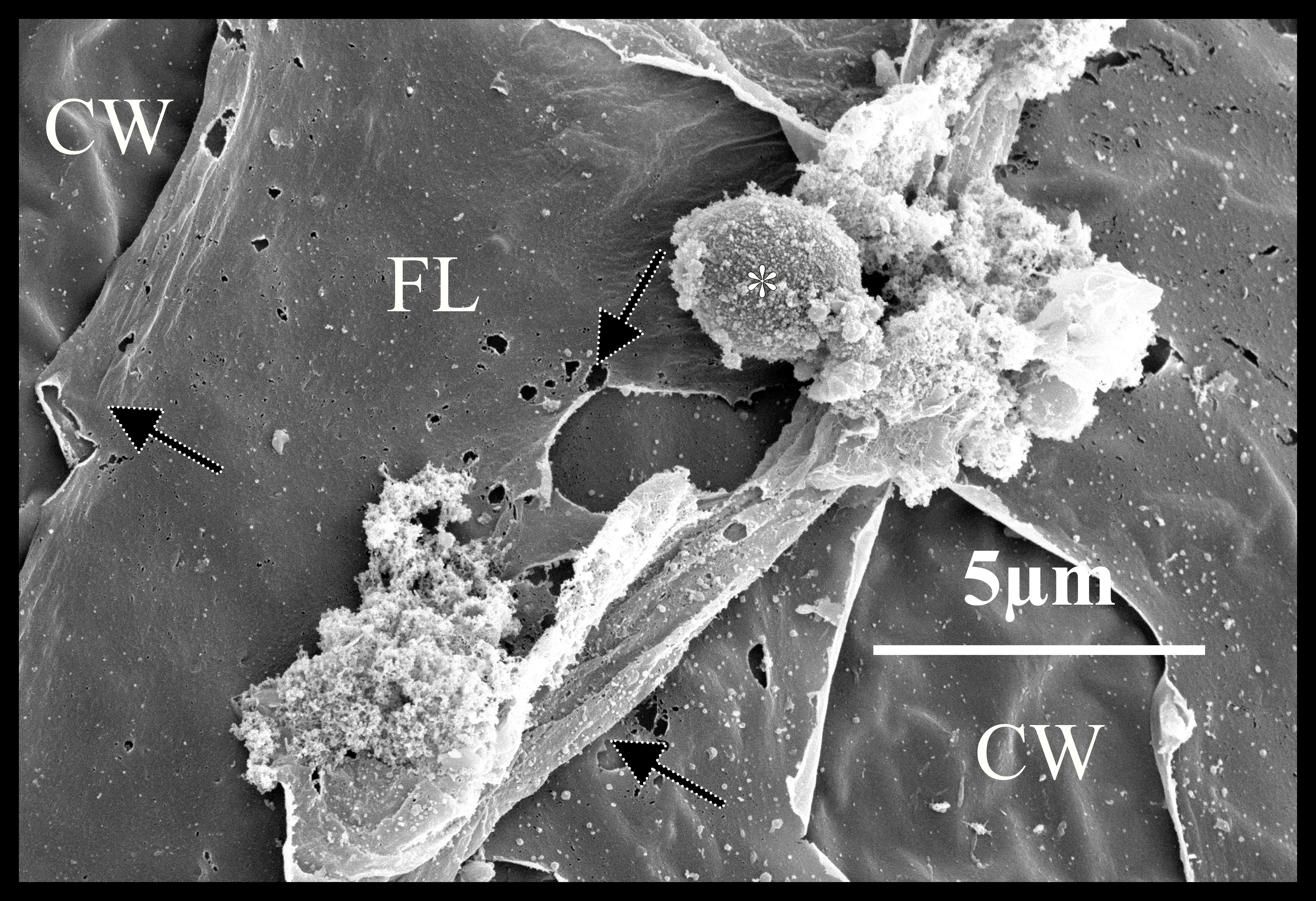

Supplement: Supplementary file 3 — Figure S2. Additional flexible layer (FL) observed between the cyst wall (CW) ‘outer layer’ and spongey layer folds, tears and twists (arrows). *, unidentified microbes. [file EMI4-16-e70035-s005.tif]
